# An Annotated Chromosome-Level Reference Genome of the Red-Eared Slider Turtle (*Trachemys scripta elegans*)

**DOI:** 10.1093/gbe/evaa063

**Published:** 2020-04-06

**Authors:** Warren Brian Simison, James F Parham, Theodore J Papenfuss, Athena W Lam, James B Henderson

**Affiliations:** e1 Center for Comparative Genomics, California Academy of Sciences, San Francisco, California; e2 Department of Geological Sciences, California State University Fullerton; e3 Museum of Vertebrate Zoology, University of California, Berkeley

**Keywords:** reference genome, Hi-C, linked-reads, IsoSEQ, turtle, synteny, chromosome, assembly

## Abstract

Among vertebrates, turtles have many unique characteristics providing biologists with opportunities to study novel evolutionary innovations and processes. We present here a high-quality, partially phased, and chromosome-level Red-Eared Slider (*Trachemys scripta elegans*, TSE) genome as a reference for future research on turtle and tetrapod evolution. This TSE assembly is 2.269 Gb in length, has one of the highest scaffold N50 and N90 values of any published turtle genome to date (N50 = 129.68 Mb and N90 = 19 Mb), and has a total of 28,415 annotated genes. We introduce synteny analyses using BUSCO single-copy orthologs, which reveal two chromosome fusion events accounting for differences in chromosome counts between emydids and other cryptodire turtles and reveal many fission/fusion events for birds, crocodiles, and snakes relative to TSE. This annotated chromosome-level genome will provide an important reference genome for future studies on turtle, vertebrate, and chromosome evolution.

## Introduction

The application of whole-genome sequencing to non-model organisms is providing new insights into the genome evolution of tetrapods (Shedlock et al. 2007; Ellegren 2014). Because turtles (Testudines) are one of the three main groups of reptiles, they represent an important lineage for comparison. Within turtles, studies of genomic evolution have contributed to a broader understanding of many turtle questions, including sex determination mechanisms (Bachtrog et al. 2014; Montiel et al. 2016; Platt et al. 2017; Lee et al. 2019). Turtle genomes are also important because turtles are renowned for their ability to hybridize across distantly related lineages (Buskirk et al. 2005). The high-quality, annotated, and chromosome-level genome of the Red-Eared Slider (*Trachemys scripta elegans*, TSE hereafter) (NCBI BioProject PRJNA552319) presented here is an important source of data for future research into these and other questions relating to the evolution of tetrapod genomes. This genome assembly includes 10× linked-reads, Hi-C data (Lieberman-Aiden et al. 2009), mate-pair data, and PacBio Iso-Seq data. We chose to sequence TSE for this study because it is the most abundant turtle on Earth, has a long history as a comparative subject in turtle studies (Gibbons 1990), and is of conservation and evolutionary interest as a hybridizing introduced species (Parham et al. 2013, 2020).

We apply Benchmarking Universal Single-Copy Ortholog v2.0.1 (BUSCO) synteny analyses to the TSE genome compared with two other turtles (a tortoise and a sea turtle) and three other diapsids (alligator, chicken, and python) and identify patterns of chromosomal fission and fusion events across these diapsids.

## Materials and Methods

### DNA and RNA Sample Collection

DNA extractions for the 10× genomics linked-reads and RNA extractions for the PacBio Iso-Seq libraries were from freshly collected liver tissue sent to Genewiz (www.genewiz.com, last accessed April 8, 2020) for high-molecular weight DNA extractions. The mate-pair library was from freshly collected liver tissue from a different TSE specimen (CAS 252980) and performed by NGX Bio (ngxbio.com, last accessed April 8, 2020). DNA extraction for the Hi-C library was performed in the Center for Comparative Genomics at the California Academy of Sciences from 200 mg of freshly harvested liver tissue of a TSE from Texas (MVZ 292727) (For more detail on library preparation and sequencing, see supplementary text, Supplementary Material online).

### Genome Assembly

Our de novo assembly began with the assembly of the 10× reads using Supernova release 2.0.1 (Weisenfeld et al. 2017) to generate a draft genome assembly. We began with a library of 429 M read pairs of Illumina 2×150 data barcoded by the 10× Genomics Chromium instrument.

Next, we used the 4-kb insert mate-pair reads, adapter trimmed and classified by Illumina’s NxTrim (O’Connell et al. 2015), to scaffold the Supernova generated assembly with BESST version 2.2.8 (Sahlin et al. 2014, 2016); default parameters were used except opts=“–iter 20000000” which sets a maximum of 20 million iterations.

The third step was to use ARKS v1.0.2 (Coombe et al. 2018), which is an alignment-free assembler using a k-mer-based mapping approach for 10× linked-read data. ARKS reuses the original 10× Illumina reads for k-mer mapping against the Supernova/BESST assembly in three steps. The first step uses a k-mer approach to map the linked barcodes to the Supernova/BESST contigs. ARKS then scores contig pairs, and finally, it produces a scaffold graph with estimated distances. The companion LINKS program (Warren et al. 2015) applies this graph to create the longer scaffolded assembly.

We then incorporated the Hi-C data for super-scaffolding using the Proximo assembly pipeline, performed by Phase Genomics. Chromatin conformation capture data were generated using a Phase Genomics (Seattle, WA) Proximo Hi-C Animal Kit, which is a commercially available version of the Hi-C protocol (Lieberman-Aiden et al. 2009). Following the manufacturer’s instructions for the kit, intact cells from two samples were crosslinked using a formaldehyde solution, digested using the Sau3AI (*Mbo*I) restriction enzyme, and proximity ligated with biotinylated nucleotides to create chimeric molecules composed of fragments from different regions of the genome that were physically proximal in vivo, but not necessarily genomically proximal. Molecules were pulled down with streptavidin beads and processed into an Illumina-compatible sequencing library. Sequencing was performed on an Illumina HiSeq4000, generating a total of 442,350,436 PE150 read pairs.

Briefly, reads were aligned using BWA-MEM (Li and Durbin 2009) with the -5SP and -t 8 options specified, all others default. SAMBLASTER (Faust and Hall 2014) was used to flag PCR duplicates, which were later excluded from analysis. Alignments were then filtered with SAMtools (Li et al. 2009) using the -F 2304 filtering flag to remove nonprimary and secondary alignments.

The Phase Genomics’ Proximo Hi-C genome scaffolding platform was used to create chromosome-scale scaffolds from the corrected assembly as described in Bickhart et al. (2017). As in the LACHESIS method (Burton et al. 2013), this process computes a contact frequency matrix from the aligned Hi-C read pairs, normalized by the number of Sau3AI restriction sites (GATC) on each contig, and constructs scaffolds in such a way as to optimize expected contact frequency and other statistical patterns in Hi-C data. Approximately 100,000 separate Proximo runs were performed to optimize the number of scaffolds and scaffold construction in order to make the scaffolds as concordant with the observed Hi-C data as possible.

We then ran SOAP GapCloser version 1.12 (Luo et al. 2012) with barcode and adapter trimmed 10× paired-reads together with the mate-pair reads for gap closing; parameters -l 152 -p 31 were used. This was followed by a decontamination and duplicate identification step using NCBI’s tbl2asn script, which generates a “.val” error file listing potential contaminants, mitochondrial sequences, and duplicates. We used this information to manually remove contaminants and duplicates identified by tbl2asn. This step also serves to prepare the sequence data for easier submission to GenBank. Along with the removal of exact duplicates identified by tbl2asn, we also removed near-duplicate contigs that differed by a small number of bases from each other and records with 90% or more Ns; presumably, these are an artifact of the Supernova program’s attempt to phase the genome. This was followed by two more SOAP GapCloser runs.

We made manual Hi-C scaffold adjustments with Juicebox (Durand et al. 2016) (supplementary text and fig. S1, Supplementary Material online) and by using BUSCO v2.01 (Simao et al. 2015) with the 3,950 ortholog Tetrapoda odb9 database for synteny comparisons of TSE with other Archelosauria (supplementary table S1, Supplementary Material online, exclusive of *Python*). This assembly is named Tse_1.0.fasta (NCBI BioProject PRJNA552319).

To assess the quality and completeness of our assembly, we used BUSCO. We employed the reference gene set of Tetropoda odb9 (a total of 3,950 orthologs) and ran the genome option of the program using the –limit 20 parameter. We also ran BUSCO with the same parameters on a reverse complement of the assembly (For details on genome size estimation see supplementary text, Supplementary Material online).

### Genome Annotation and Analyses

#### Repeat Analysis

For TSE and three other turtles’ assemblies, *Chelonia mydas, Chrysemys picta bellii*, and *Gopherus evgoodei*, we created a species specific de novo repeat library file by running RepeatModeler version open-1.0.11 (Smit and Hubley 2008–2015 www.repeatmasker.org, last accessed April 8, 2020). RepeatModeler marshals RECON version 1.08 (Bao and Eddy 2002), RepeatScout version 1.0.5 (Price et al. 2005), and Tandem repeats finder version 4.09 (Benson 1999) to create a de novo repeat library. We ran RepeatMasker on each assembly with options -nolow and -lib referencing a custom library combining its de novo repeat families with the vertebrate RepBase Combined Database (Dfam_3.0 from RepeatMasker and RepBase-20181026 input to rmblastn version 2.9.0+).

We annotated the TSE assembly using Maker version 3.01.02 (Holt and Yandell 2011; Campbell et al. 2014) to predict gene models and predict functional annotations. We ran MAKER in two runs with both homology-based and ab initio gene modelers (for complete details see supplementary text, Supplementary Material online).

#### BUSCO Synteny Analyses

The term “synteny” has been applied to different types of genetic patterns (Renwick 1972; Passarge et al. 1999). We use the term “synteny” sensu Shields (2001), “conservation of order of orthologous genes between different species.” In order to analyze synteny among lineages, we used the results of BUSCO analyses and custom scripts (github.com/calacademy-research/ccgutils/tree/master/assembly_scripts, last accessed April 8, 2020) to generate single-copy ortholog (SCO) Circos (Krzywinski et al. 2009) synteny “links” files between TSE and two other turtles (a tortoise, *G. evgoodei* and a sea turtle, *C. mydas*), the chicken (*Gallus*), a crocodilian (*Alligator*), and a snake (*Python*) (supplementary table S1, Supplementary Material online). Although we have a comprehensive annotation of the TSE genome, many available genomes are not annotated and cannot be included in synteny analyses based on annotations; or, unlike these chromosomal level assemblies, are so fragmented that the synteny matches are not insightful. Because BUSCO analyses can be run relatively easily and quickly (∼24 h) relative to a full annotation (typically weeks), we propose and demonstrate the utility of BUSCO-based synteny analyses. We chose genomes with high-quality chromosome-level assemblies because synteny analyses are sensitive to fragmented assemblies (Liu et al. 2018). For *G. evgoodei*, we are uncertain of the karyotype; the 24 reported scaffolds likely reflect chromosomes, but further assembly could identify additional smaller chromosomes. Several studies (reviewed by Bickham and Carr 1983) show that most testugurians (the group that includes *G. evgoodei* and other tortoises) are 2*n* = 52 and that this condition is likely ancestral for that clade (Bickham and Carr 1983).

## Results and Discussion

### Genome Assembly

Proximo Hi-C scaffolding resulted in an initial set of additional scaffolds, with which Juicebox and BUSCO synteny analysis with four other archelosaurs were used to correct scaffolding errors as well as introduce eight new breaks into putative misjoined scaffolds from the original assembly. A total of 414 scaffolds were placed and oriented by these methods into 27 new scaffolds; 395 of these comprise the 25 haploid chromosomes of TSE, and 19 of these were used in two unplaced scaffolds.

The resulting Tse_1.0 assembly is 2.269 Gb in length, where the GenomeScope (Vurture et al. 2017) k-mer frequency estimate was just over 2 Gb and Supernova estimated 2.41 Gb (supplementary fig. S2, Supplementary Material online). This assembly size is consistent with other published turtle genome’s sizes (supplementary table S2, Supplementary Material online), the average of which for six assemblies is 2.33 Gb. Tse_1.0 scaffold N50 is 129.68 Mb occurring at chromosome 6 (chr1–chr6 contain 54.84% of the bases) and 19-Mb scaffold N90 at chr21 (chr1–chr21 contain 90.51% of total bases). Contig N50 is 189,165 bp; contig N90 is 32,113 bp. The 25 assembled haploid chromosomes contain 92.92% of the full assembly leaving 7.08% currently unplaced. This is one of the highest scoring turtle genomes published to date (supplementary table S2, Supplementary Material online). We used a modified Assemblathon script (github.com/calacademy-research/ccgutils/tree/master/asmstats, last accessed April 8, 2020) to calculate TSE assembly statistics (table 1).

**Table 1:** Assemblathon+ Statistics for TSE Genome Assembly (generated with custom asmstats.pl^a^)

Number of scaffolds >1K nt	26,710	66.60%	
Number of scaffolds >10K nt	2,988	7.50%	
Number of scaffolds >100K nt	32	0.10%	
Number of scaffolds >1M nt	28	0.10%	
Number of scaffolds >10M nt	24	0.10%	
Mean scaffold size	56,571		
Median scaffold size	1,555		
N50 scaffold length	129,675,691	L50 scaffold count	6
N60 scaffold length	126,808,733	L60 scaffold count	7
N70 scaffold length	85,829,911	L70 scaffold count	10
N80 scaffold length	43,716,676	L80 scaffold count	13
N90 scaffold length	19,049,219	L90 scaffold count	21
Scaffold %A	27	Number of A	609,556,304
Scaffold %C	21	Number of C	482,905,406
Scaffold %G	21	Number of G	483,034,848
Scaffold %T	27	Number of T	609,529,607
Scaffold %*N*	4	Number of *N*	83,700,144
Scaffold %non-ACGTN	0		
Number of scaffold non-ACGTN nt	0		
Percentage of assembly in scaffolded contigs	94.50		
Percentage of assembly in unscaffolded contigs	5.50		
Average number of contigs per scaffold	1.5		
Average length of break (≥10 *N*) between contigs in scaffold	4,165		

Number of contigs	60,193		
Number of contigs in scaffolds	22,314		
Number of contigs not in scaffolds	37,879		
Total size of contigs	2,185,039,207		
Longest contig	1,642,093		
Shortest contig	48		
Number of contigs >1K nt	45,044	74.80%	
Number of contigs >10K nt	19,706	32.70%	
Number of contigs >100K nt	6,735	11.20%	
Number of contigs >1M nt	21	0.00%	
Number of contigs >10M nt	0	0.00%	
Mean contig size	36,301		
Median contig size	3,258		
N50 contig length	189,165	L50 contig count	3,255
N60 contig length	146,678	L60 contig count	4,570
N70 contig length	108,417	L70 contig count	6,295
N80 contig length	71,323	L80 contig count	8,775
N90 contig length	32,113	L90 contig count	13,220
Contig %A	28	Number of A	609,556,304
Contig %C	22	Number of C	482,905,406
Contig %G	22	Number of G	483,034,848
Contig %T	27.9	Number of T	609,529,607
Contig %N	0	Number of N	13,042
Contig %non-ACGTN	0		
Number of contig non-ACGTN nt	0		

^a^ asmstats.pl is a modification of github.com/ucdavis-bioinformatics/assemblathon2-analysis/blob/master/assemblathon_stats.pl (last accessed April 8, 2020) available at github.com/calacademy-research/ccgutils/tree/master/asmstats (last accessed April 8, 2020).

### Genome Annotation and Analyses

#### Genome Annotation

After the MAKER runs and InterProScan results, we determined the TSE set of 28,415 predicted gene models in the assembly and added functional annotations to them based on homology results.

We assigned homology-based annotation to 27,439 (96.33%) of the proteins for which the genes code, with 57.26% of those assigned to a *Chrysemys picta bellii* homolog and a total of 86% (23,883) assigned to homologs of one of five turtle species. About 28,903 Pfam domains were found with 6,039 of them unique; 43,835 Gene Ontology (GO) terms, 3,703 unique; 77,913 InterPro matches, 14,749 unique. Pathways from three pathway databases show 3,287 KEGG pathways, 904 unique; 3,179 MetaCyc, 622 unique; and 27,381, 1,606 unique, from the Reactome database.

#### Repeat Analysis

The results from the RepeatMasker analyses for TSE, *C. mydas*, *Chrysemys picta belli*, and *G. evgoodei* are listed in supplementary table S3, Supplementary Material online.

#### Quality Assessment

The BUSCO analyses were run twice, in the forward direction and on the reverse complement. The reverse complement run produced an additional 29 complete and 1 fragmented BUSCO, thus reducing missing ones by 30 from the forward run. About 95.8% complete BUSCOs were found, 3,783 with 25 duplicates, 106 fragmented, and 61 missing from the reference gene set 3,950 in Tetropoda odb9 (supplementary table S4, Supplementary Material online).

### Comparative Genomic Analyses

#### BUSCO Synteny

We mapped shared orthologs from the Tetrapoda odb9 database of BUSCOs to each of the genomes used in this study (TSE, *G. evgoodei*, *C. mydas*, *Alligator mississippiensis*, *Gallus gallus*, and *Python bivittatus*) and created Circos links files to generate Circos synteny diagrams (supplementary figs. S4–S6, Supplementary Material online). We demonstrate here the utility of BUSCO synteny analyses by identifying clear chromosomal fission/fusion events and patterns (creation and loss of chromosomes) across the diapsids (squamates, birds, crocodiles, and turtles).

##### Chromosome Fission/Fusion

**Fig. 1:**
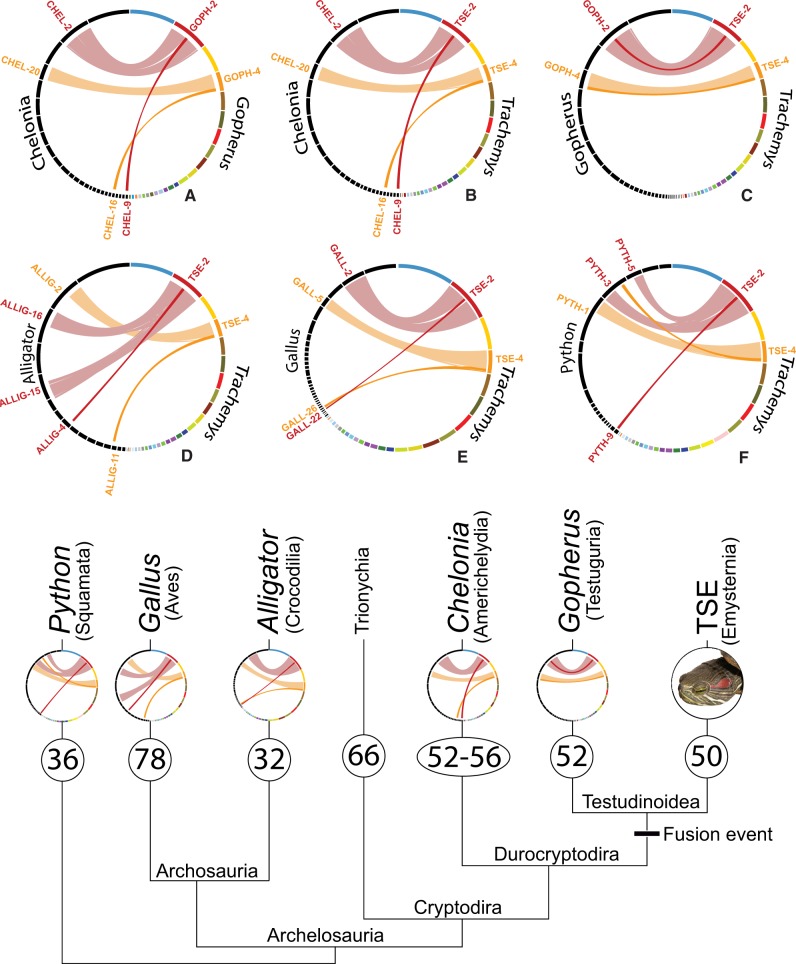
—Circos synteny diagrams displaying chromosomal fissions/fusions within diapsids. The dark red and dark orange lines represent homologous clusters of SCOs found on chromosomes not found on testudinoid genomes (TSE and *Gopherus*) and likely fused with TSE and *Gopherus* chromosomes 2 and 4. Synteny diagram (*A*) reveals the fusion of two *Chelonia* chromosomes 16 and 9 with *Gopherus* chromosomes 2 and 4 respectively. Synteny diagram (*B*) reveals the fusion of two *Chelonia* chromosomes 16 and 9 with TSE chromosomes 2 and 4 respectively. Synteny diagram (*C*) reveals that for both *Gopherus* and TSE the dark red and dark orange cluster of SCOs have fused with TSE and *Gopherus* chromosomes 2 and 4. Synteny diagram (*D*) and (*E*) reveal the archosaurian *Alligator* chromosomes 4 and 11 and *Gallus* chromosomes 26 and 22 have fused with testudinoid (*Gopherus* and TSE) chromosomes. Synteny diagram (*F*) reveals that *Python* chromosome 9 has fused with *Gopherus* and TSE chromosome 2. The dark orange cluster of SCOs from TSE chromosome 4 are part of *Python* chromosome 5. The colored bars in rings represent TSE chromosomes except in (*A*), where colored bar represent *Gopherus*. Black bars represent other chromosomes. Note that all single relocations have been removed for clarity. For the phylogenetic tree of reptile and avian genomes used in this study, the number in ovals represents the hypothesized ancestral 2*n* number of chromosome for each lineage based on Bickham and Carr (1983) and the circular diagrams represent the synteny diagrams highlighting the chromosomes involved in the indicated fusion event. TSE and *Gopherus* share the same fusion of these two clusters (diagram *C*).

Using BUSCO synteny analyses, we were able to identify various chromosomal fission/fusion events and the lineages where they occurred. We identify two clusters of BUSCOs shared among all diapsids examined in this study. For TSE and *Gopherus*, these BUSCO clusters are part of two pair of homologous chromosomes TSE-2 to GOPH-2, and TSE-4 to GOPH-4 (fig. 1*C*), for all other taxa, these clusters are found on different and nonhomologous chromosomes (fig. 1*A*, *B*, and *D*–*F*). Of the archelosaurian genomes studied here, only *Alligator* has fewer chromosomes (32) than TSE and *Gopherus* (50 and 52 respectively, supplementary table S1, Supplementary Material online), which suggests that two chromosomal fusions occurred sometime since the most recent common ancestor of Testudinoidea (fig. 1 tree).

The genomic evolution of birds has produced genomes with many small chromosomes (Burt 2002). The syntenic comparison between TSE and *Gallus* reveals at least six fusion events relative to TSE (supplementary fig. S5*A*, Supplementary Material online), which accounts for six additional chromosomes for *Gallus* relative to TSE. In contrast to birds, crocodiles have a reduced number of chromosomes (*Gallus* [2*n* = 78], *Alligator* [2*n* = 32]). We see seven clear fission events relative to TSE and account for a greater number of chromosomes in TSE (supplementary fig. S5*B*, Supplementary Material online). All six of the TSE chromosomes involved in fission/fusion with *Gallus* are shared by the *Alligator* versus TSE analysis. The only exception is the addition of TSE chromosome 1 (TSE-1) in the *Alligator* versus TSE analysis (supplementary fig. S5*B*, Supplementary Material online). A BUSCO synteny analysis between the squamate *Python* (2*n* = 36) and TSE reveals more than a dozen clear fission/fusion events relative to TSE. *Python* chromosomes 1, 2, and 3 each appear to be comprised at least four large syntenic blocks from separate TSE chromosomes (supplementary fig. S6, Supplementary Material online). TSE chromosomes 2, 4, 8, 9, and 10 each have large syntenic blocks that map to two different *Python* chromosomes (supplementary fig. S6, Supplementary Material online).

## Supplementary Material

evaa063_Supplementary_Data
