# Supplementary material for: An Annotated Chromosome-Level Reference Genome of the Red-Eared Slider Turtle (Trachemys scripta elegans)
Source: Genome Biol Evol. 2020 Apr 6;12(4):456–62. doi: 10.1093/gbe/evaa063 (PMC7186784; doi:10.1093/gbe/evaa063)
Supplement: evaa063_Supplementary_Data [file evaa063_supplementary_data.pdf]

Supplementary Material for:

An Annotated Chromosome-Level Reference Genome of the Invasive Red-eared Slider Turtle

W. Brian Simison[1*], James F. Parham[1,2], Theodore J. Papenfuss[3], Athena W. Lam[1], James B. Henderson[1]

[1]Center for Comparative Genomics, California Academy of Sciences, 55 Music Concourse Dr. San Francisco, CA 94188
[2]California State University Fullerton, Fullerton, CA 92834
[3]Museum of Vertebrate Zoology, 3101 Valley Life Sciences Building, University of California, Berkeley, CA 94720
[*]Author for Correspondence: W. Brian Simison, Center for Comparative Genomics, California Academy of Sciences, 55 Music Concourse Dr., San Francisco, CA 94188, bsimison@calacademy.org

Supplemental Material

Materials and Methods

Libraries and sequencing

Library preparation and sequencing for 10x Genomics linked-reads, and PacBio Iso-Seq were outsourced to Genewiz. Mate-pair libraries were prepared and sequenced by NGX Bio.

For the Hi-C library, we used the Hi-C Animal kit from Phase Genomics (https://phasegenomics.com) for chromatin isolation and library preparation according to manufacturer's protocol. The Hi-C libraries were sequenced on one lane of Hiseq 4000 (> 400M paired-end reads at 150bp).

Hi-C scaffold adjustments with Juicebox (Durand et al. 2016)

Manual modification of the Hi-C assembly using Juicebox v 1.11.08.

We used Juicebox to make minor adjustments to the Hi-C assembly. In Juicebox, we opened the .hic file generated by Juicer then imported the initial. assembly file generated by Juicer. Using the available tools in Juicebox, we made slight modifications to the assembly based on our SCO synteny analyses and based on observable contact patterns in Juicebox then saved that assembly as our final assembly file. To visualize the final assembly in Juicebox, we repeated the loading of the .hic file, importing the initial assembly, then imported the final modified assembly with the "Import Modified Assembly" under the "Assembly" menu.

FIG. s1.

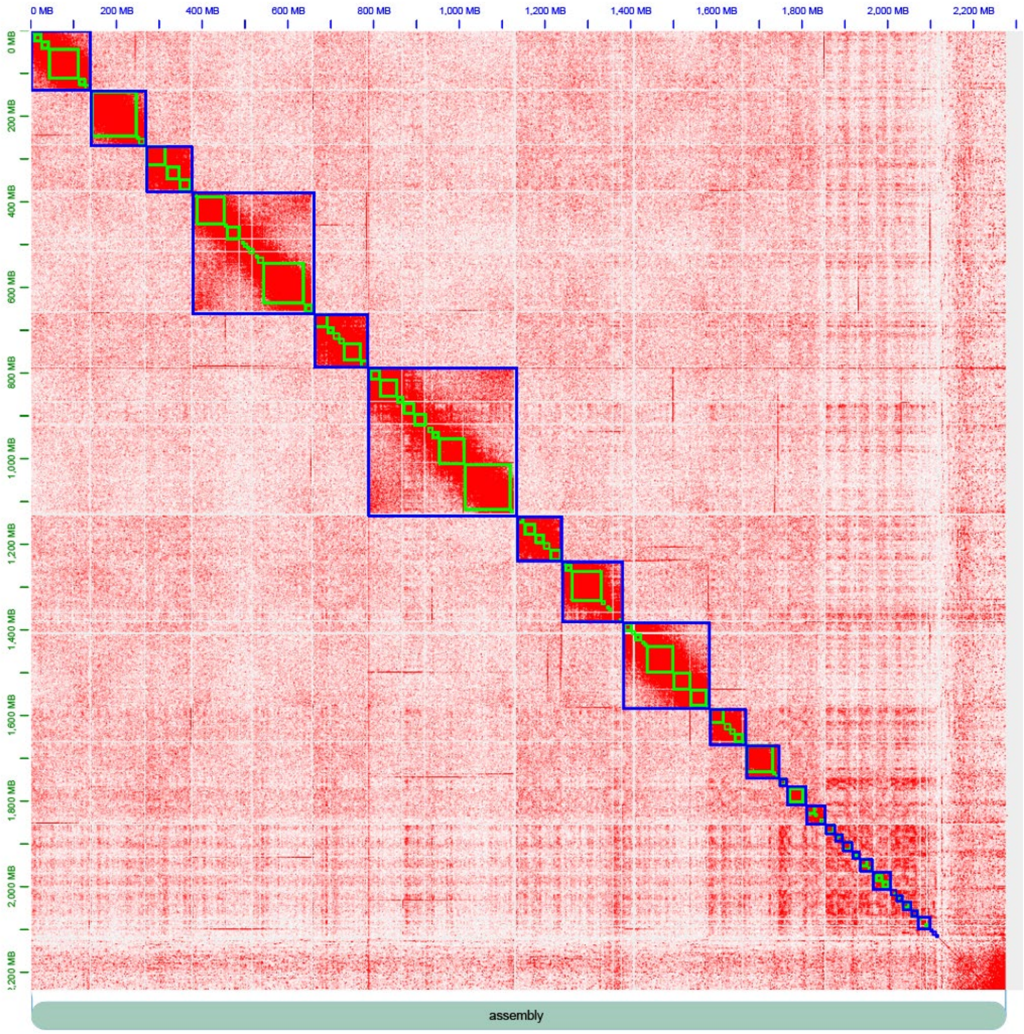

Hi-C contact map. The final visualization of the TSE Hi-C assembly. The axes of the diagram are the genomic coordinates of the TSE assembly mapped against itself. Red color intensity

reflects the Hi-C contact frequency between regions of the assembly. Blue boxes represent assembled chromosomes and green boxes represent assembled scaffolds that comprise the chromosome.

Table s1.
List of genomes used in synteny analyses, their reported (or estimated) number of chromosomes, and associated URLs

Species	Common Name	# Chromosomes (2N)	Reference
Gopherus evgoodei	Goode's Desert Tortoise	≥48*	Vertebrate Genome Project (Koepfli et al. 2015)
Chelonia mydas	Green Sea Turtle	56	(Wang et al. 2013; Dudchenko et al. 2017, 2018)
Alligator mississippiensis	American Alligator	32	(Rice et al. 2017; Dudchenko et al. 2017, 2018; St John et al. 2012)
Gallus gallus	Chicken	72	(Hillier, et al. 2004)
Python bivittatus	Burmese Python	36	(Castoe et al. 2013; Dudchenko et al. 2017, 2018)

*unpublished methods, chromosome count may eventually be more.

Genome Size Estimate

We quality trimmed and removed the barcodes of the 10x linked-reads then counted their k-mers of length 21 and created a k-mer frequency histogram with Jellyfish v.2.3.0 (Marçais & Kingsford 2011). This file was uploaded to GenomeScope (Vurture et al. 2017) for analysis. The 10x Genomics' Supernova genome assembly program we also used to reports a size estimate. We also used the k-mer analysis tool KAT (Mapleson et al. 2017) to evaluate and characterize the TSE assembly for heterozygosity, size, completeness, sequence bias, and coverage (fig. s3). GenomeScope estimate of a little over 2 Gb and Supernova's 2.41 Gb yields a value similar to six other turtle genome which have a median genome assembly size of 2.33 Gb (table s2).

Gene Model Prediction and Functional Annotation

The first step is to identify and mask repetitive and transposable elements in the assembly with RepeatMasker (http://www.repeatmasker.org/) and RepeatRunner because they interfere with downstream ab initio gene prediction algorithms. For the first run, we specified `model_org=vertebrates` in the MAKER ctl file so RepBase masking in RepeatMasker runs with `-species vertebrate` and also specified `rmlib=TSE-families.fa` for MAKER to additionally apply the custom repeat library created for TSE by RepeatModeler.

Masking was followed by ab initio gene prediction using SNAP (Korf 2004), trained with TSE single copy BUSCOs, Augustus (Stanke et al. 2006, 2008) with defaults, and GENSCAN (Burge & Karlin 1997) self-trained with the TSE assembly. For homology predictions, we used TSE Iso-Seq transcripts, TSE single copy BUSCO transcripts, and GenBank's mRNA sequences for TSE; an additional set of transcripts was provided with all *Chrysemys picta bellii* mRNA sequences found in GenBank. Protein homology evidence was provided by protein sequences from the *Chrysemys picta bellii* assembly (GCA_000241765), *Gopherus agassizii* (Tollis et al. 2017), *Chelonoidis abindgonii* aka Lonesome George (lgeorge.augustus.func_protein.fa in github.com/vqf/LG), and Uniprot-SwissProt vertebrate proteins.

For the second run, SNAP trained with the gene models found in the first MAKER run, Augustus trained from this SNAP output, and FGENESH (Solovyev et al. 2006) with the *Chrysemys picta bellii* training set were used as ab initio predictors.

InterProScan v5.36-75.0 was run on the output protein sequences from the second MAKER run to annotate Pfam domains of this preliminary gene model set. Gene models having a Pfam protein domain, Interpro match or annotated estimated distance (AED) < 1.0 were designated as the TSE standard gene model set. Additionally, tRNAscan-SE v2.0.2 was run to discover tRNAs in the genome assembly.

For functional annotation, blastp was run against GenBank NR, Uniprot-SwissProt, and Uniprot-TrEMBL; blastn was run against the GenBank NT database with the best scoring match chosen as the functional annotation.

Genome Subscaffold Statistics

As a measure of the quality before the chromosomal level assembly was created with the Hi-C data, we broke the Hi-C scaffolds into their constituent scaffolds and reran the statistics. This pre Hi-C version has N50 23.87 Mb L50 23, N90 956,351 bp L90 169, indicating a high level of quality prior to applying Hi-C data and toolsets which used 414 of these input scaffolds to create 27 larger scaffolds.

FIG. s2.

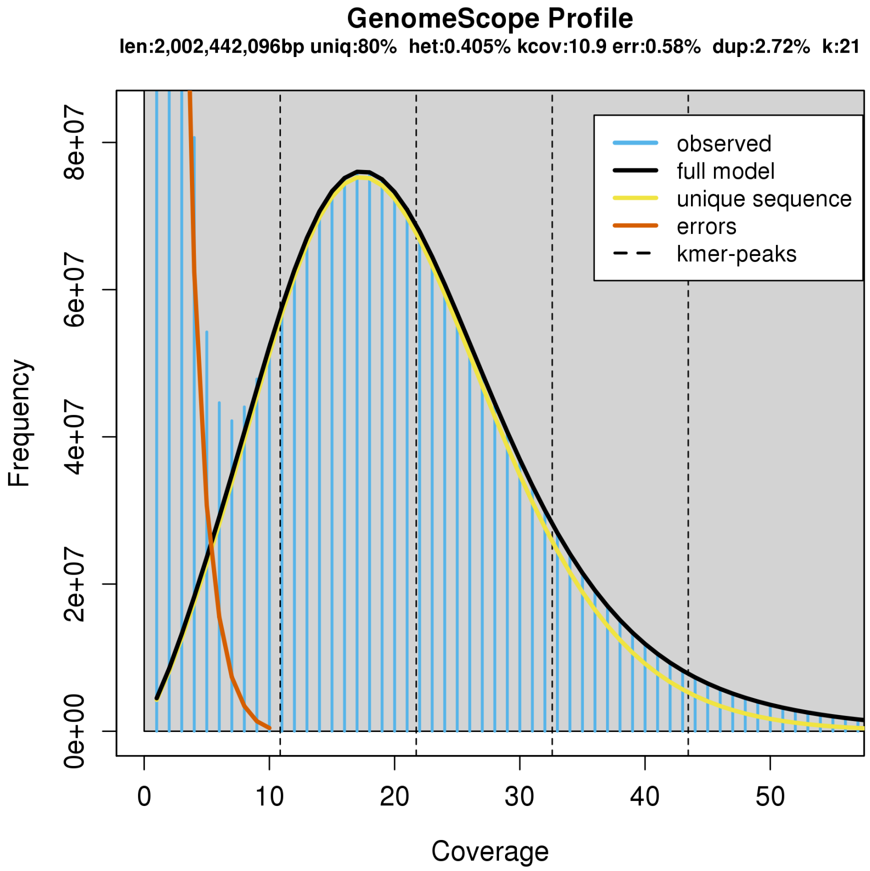

The GenomeScope profile.

FIG. s3.

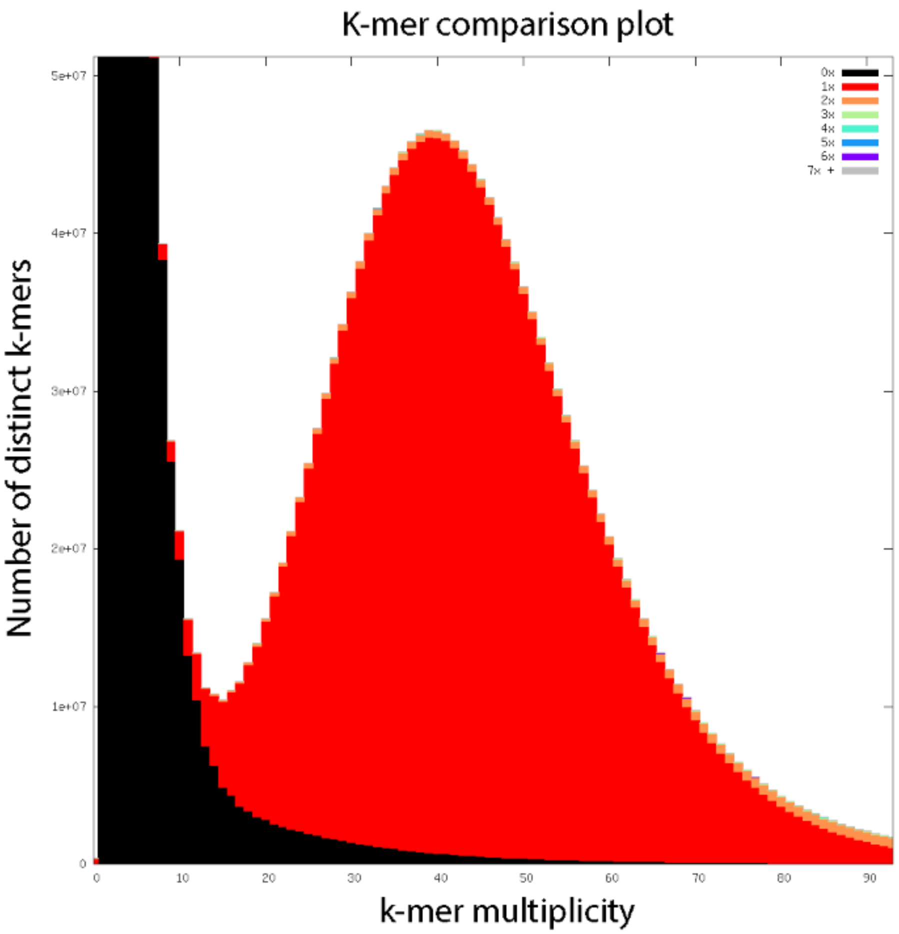

KAT k-mer analysis spectra-cn plot. This spectra-cn plot represents how many elements of each frequency on the read's spectrum ended up not being included in the assembly, included once, included twice etc.

Table s2.
Genome assembly statistics for published turtle genomes

	Pelodiscus sinensis	***Chelonia mydas***	***Gopherus agassizii v2***	***Gopherus evgoodei***	***Platysternon megacephalum***	***Chrysemys picta bellii***	**TSE**
**Assembly size (Gb)**	2.21	2.24	2.34	2.29	2.32	2.29	2.26
**Sequence coverage (×)**	105.6	82.3	118	58	204.2	58	53.5
**Contig N50 (kb)**	21.9	20.4	42.46	13,026	41.8	13,026	189.16
**Scaffold N50 (kb)**	3,331	3,778	28,360	147,425	7,222	147,425	129,680
**GC content (%)**	44.4	43.5	43	44.2	44.63	44.2	44.2
**Gene number**	19,327	19,633	25,469	26,465	22,400	26,465	28,425

Table s3.
Summary of GC and Repeat content from the repeat analyses. Each RepeatMasker run used a library consisting of exported RepBase vertebrate repeats combined with the species' RepeatModeler de novo repeat set.

	Chelonia mydas		***Gopherus evgoodei***		***Chrysemys picta belli***		**TSE**	
**Type**	**Length (bp)**	**Genome %**	**Length (bp)**	**Genome %**	**Length (bp)**	**Genome %**	**Length (bp)**	**Genome %**
**GC %**	-	43.48%	-	43.48%		44.19%		44.21%
**SINEs:**	50,606,912	2.29%	50,606,912	2.29%	51,665,281	2.18%	52,136,789	2.30%
**LINEs:**	271,559,338	12.29%	271,559,338	12.29%	272,406,219	11.51%	286,249,728	12.61%
**LTR elements:**	216,684,587	9.81%	216,684,587	9.81%	225,430,485	9.53%	213,952,833	9.42%
**DNA elements:**	324,345,284	14.68%	324,345,284	14.68%	393,606,818	16.64%	381,028,614	16.78%
**Unclassified:**	45,511,358	2.06%	45,511,358	2.06%	38,208,902	1.62%	75,362,182	3.32%
**Total interspersed repeats:**	908,707,479	41.13%	908,707,479	41.13%	981,317,705	41.48%	1,008,730,146	44.43%
**Small**	9,927,858	0.45%	9,927,858	0.45%	9,599,586	0.41%	10,776,830	0.47%
**Satellites:**	1,934,865	0.09%	1,934,865	0.09%	2,663,878	0.11%	2,310,756	0.10%
**Total**	**920,570,202**	**41.67%**	**920,570,202**	**41.67%**	**993,581,169**	**42.00%**	**1,021,817,732**	**45.00%**

Table s4.
Single Copy Ortholog completeness statistics from BUSCO analysis.

**Complete [Single, Duplicate]**	**Fragmented**	**Missing**	**Total orthologs**
3783 [3758, 25]	*106*	*61*	*3950*
95.8% [95.1%, 0.6%]	*2.7%*	*1.5%*	*3950*

FIG. s4.

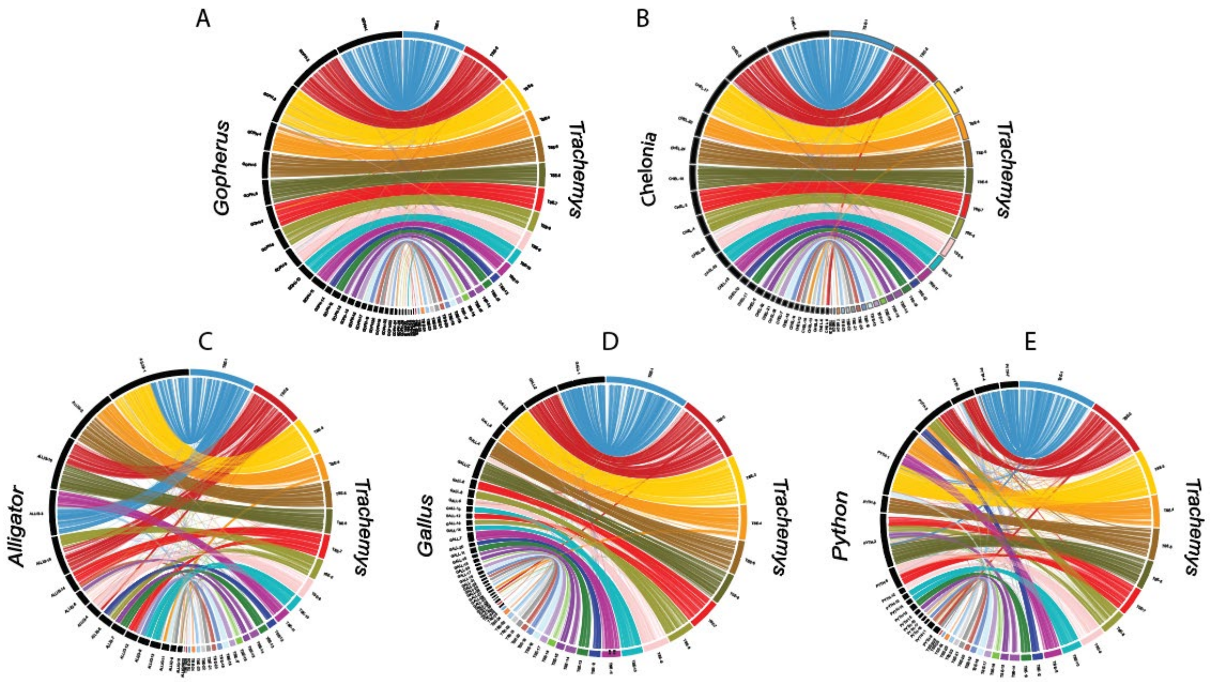

Pairwise BUSCO synteny diagrams (Circos). A) *Gopherus* v TSE. B) *Chelonia* v TSE. C) *Alligator* v TSE. D) *Gallus* v TSE. E) *Python* v TSE.

FIG. s5.

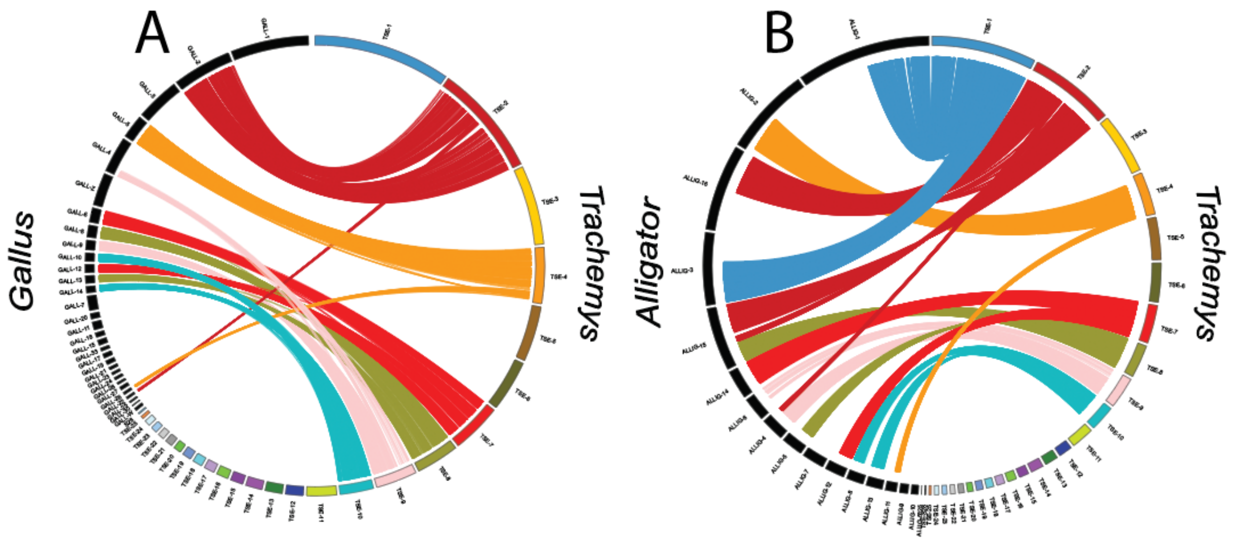

Circos BUSCO synteny diagrams showing clear fission/fusion events (A) *Gallus* vs. TSE. (B) *Alligator* vs. TSE. For *Gallus* vs. TSE, BUSCO synteny reveal six candidate fission/fusion events. For the Alligator vs. TSE BUSCO synteny reveal seven candidate fission/fusion events. Colored bars represent TSE chromosomes, black bars represent *Gallus* and *Python* chromosomes. Note that all single relocations and links associated with chromosomes not involved in obvious fission/fusion events have been removed for clarity.

FIG. s6

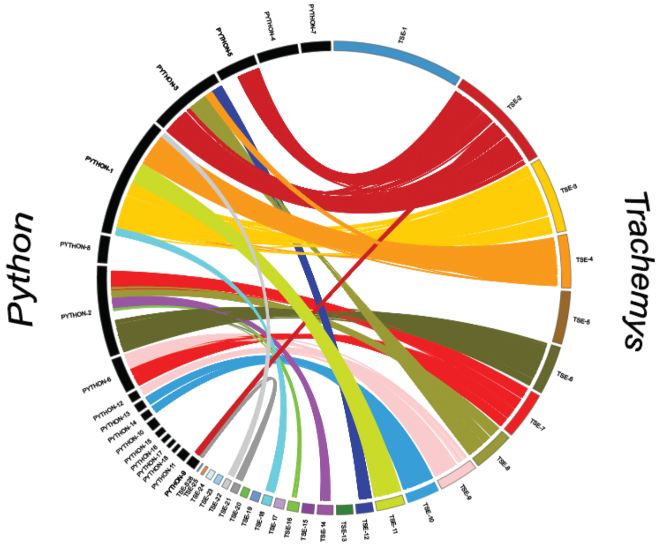

BUSCO synteny diagram between *Python* and TSE reveals more than a dozen fission/fusion events. Colored bars represent TSE chromosomes, black bars represent *Python* chromosomes. Note that all single relocations and links associated with chromosomes not involved in obvious fission/fusion events have been removed for clarity.

Literature Cited

Burge C, Karlin S. 1997. Prediction of complete gene structures in human genomic DNA. J. Mol. Biol. 268:78–94. doi: 10.1006/jmbi.1997.0951.

Castoe TA et al. 2013. The Burmese python genome reveals the molecular basis for extreme adaptation in snakes. Proc. Natl. Acad. Sci. U. S. A. 110:20645–20650. doi: 10.1073/pnas.1314475110.

Dudchenko O et al. 2017. De novo assembly of the Aedes aegypti genome using Hi-C yields chromosome-length scaffolds. Science (80-.). 356:92–95. doi: 10.1126/science.aal3327.

Dudchenko O et al. 2018. The Juicebox Assembly Tools module facilitates de novo assembly of mammalian genomes with chromosome-length scaffolds for under $1000. bioRxiv. 254797. doi: 10.1101/254797.

Hillier, L., Miller, W., Birney E et al. 2004. Sequence and comparative analysis of the chicken genome provide unique perspectives on vertebrate evolution. Nature. 432:695–716. doi: 10.1038/nature03154.

Koepfli K-P, Paten B, O'Brien SJ. 2015. The Genome 10K Project: A Way Forward. Annu. Rev. Anim. Biosci. 3:57–111. doi: 10.1146/annurev-animal-090414-014900.

Korf I. 2004. Gene finding in novel genomes. BMC Bioinformatics. 5:59. doi: 10.1186/1471-2105-5-59.

Mapleson D, Garcia Accinelli G, Kettleborough G, Wright J, Clavijo BJ. 2017. KAT: a K-mer analysis toolkit to quality control NGS datasets and genome assemblies. Bioinformatics. 33:574–576. doi: 10.1093/bioinformatics/btw663.

Marçais G, Kingsford C. 2011. A fast, lock-free approach for efficient parallel counting of occurrences of k-mers. Bioinformatics. 27:764–70. doi: 10.1093/bioinformatics/btr011.

Rice ES et al. 2017. Improved genome assembly of American alligator genome reveals conserved architecture of estrogen signaling. Genome Res. 27:686–696. doi: 10.1101/gr.213595.116.

Solovyev V, Kosarev P, Seledsov I, Vorobyev D. 2006. Automatic annotation of eukaryotic genes, pseudogenes and promoters. Genome Biol. 7 Suppl 1. doi: 10.1186/gb-2006-7-s1-s10.

St John JA et al. 2012. Sequencing three crocodilian genomes to illuminate the evolution of archosaurs

and amniotes. Genome Biol. 13:415. doi: 10.1186/gb-2012-13-1-415.

Stanke M et al. 2006. AUGUSTUS: A b initio prediction of alternative transcripts. Nucleic Acids Res. 34. doi: 10.1093/nar/gkl200.

Stanke M, Diekhans M, Baertsch R, Haussler D. 2008. Using native and syntenically mapped cDNA alignments to improve de novo gene finding. Bioinformatics. 24:637–644. doi: 10.1093/bioinformatics/btn013.

Tollis M et al. 2017. The Agassiz's desert tortoise genome provides a resource for the conservation of a threatened species. PLoS One. 12:e0177708. doi: 10.1371/journal.pone.0177708.

Vurture GW et al. 2017. GenomeScope: fast reference-free genome profiling from short reads. Bioinformatics. 33:2202–2204. doi: 10.1093/bioinformatics/btx153.

Wang Z et al. 2013. The draft genomes of soft-shell turtle and green sea turtle yield insights into the development and evolution of the turtle-specific body plan. Nat. Genet. 45:701–706. doi: 10.1038/ng.2615.
